# Inflammation-Associated Transcriptional Remodeling of Antioxidant and Stress Signaling Pathways in First-Trimester Placenta in Maternal Overweight and Obesity

**DOI:** 10.3390/biom16071033

**Published:** 2026-07-15

**Authors:** Denise Hoch, Alejandro Majali-Martinez, Francisco Algaba Chueca, Silvija Tokic, Gernot Desoye

**Affiliations:** 1 Department of Obstetrics and Gynaecology, Medical University of Graz, 8036 Graz, Austriaalejandro.majali@universidadeuropea.es (A.M.-M.); gernot.desoye@medunigraz.at (G.D.); 2 Departamento de Medicina, Salud y Deportes, Universidad Europea de Madrid, 28670 Madrid, Spain; 3 Endometriosis and Female Reproductive Health Research Group, Faculty of Medicine and Health Sciences, Universitat Rovira i Virgili, 43201 Reus, Spain; 4 Department of Basic Medical Sciences, Universitat Rovira i Virgili, 43201 Reus, Spain; 5 Department of Paediatrics and Adolescent Medicine, Medical University of Graz, 8036 Graz, Austria; 6 Research Unit of Analytical Mass Spectrometry, Cell Biology and Biochemistry of Inborn Errors of Metabolism, Medical University of Graz, 8036 Graz, Austria

**Keywords:** placenta, maternal overweight and obesity, first trimester, oxidative stress, antioxidant enzymes, stress signaling genes, oxidative damage

## Abstract

The first trimester (FT) of pregnancy is characterized by rapid placental growth and differentiation, rendering this period sensitive to maternal metabolic influences. Maternal obesity is associated with chronic low-grade inflammation and altered redox homeostasis with implications in later cardiometabolic risk in offspring, yet its impact on early placental oxidative stress remains unclear. Here, we investigated whether maternal overweight and obesity are associated with oxidative lipid and protein damage and altered expression of antioxidant enzymes and stress signaling pathways. Placental tissue from lean (BMI < 25 kg/m^2^), overweight (BMI ≥ 25 kg/m^2^) and obese (BMI ≥ 30 kg/m^2^) non-smoking women (5–12 weeks’ gestation) was analyzed. Oxidative damage was assessed by immunoblotting for 4-hydroxynonenal and 3-nitrotyrosine, and antioxidant and stress-related transcripts were quantified using targeted mRNA arrays. Explants were exposed to increased oxygen tension or TNF-α to evaluate transcriptional responsiveness. No differences in oxidative lipid or protein modifications were detected between groups. In contrast, overweight and obesity were associated with reduced expression of selected antioxidant enzymes and transcriptional remodeling of MAPK-related genes. These changes correlated with *IL6* expression but not with oxidative damage markers. Collectively, maternal overweight and obesity are linked to inflammation-associated transcriptional adaptation in FT placenta without detectable oxidative injury.

## 1. Introduction

Maternal obesity affects approximately 21% of pregnancies worldwide [1] and is associated with a broad spectrum of adverse obstetric and fetal outcomes [2]. The placenta is considered to mediate the link between maternal metabolic and inflammatory disturbances and impaired fetal development and neonatal phenotype [3]. In particular, the first trimester (FT) of human pregnancy represents a critical window during which the placenta establishes the maternal–fetal interface through rapid trophoblast proliferation, differentiation, and vascular remodeling [4]. Initially, these processes occur in a physiologically low-oxygen environment. The onset of maternal blood flow into the intervillous space typically occurs between 8 and 12 weeks of gestation and is accompanied by a rise in local oxygen tension and reactive oxygen species (ROS) production [5,6]. While controlled levels of ROS play essential roles in placental signaling and development [6], excessive or dysregulated oxidative stress may compromise placental integrity and has been linked to early pregnancy failure [7]. Indeed, the early placenta is particularly susceptible to oxidative stress because of its intrinsically low antioxidant capacity and its reliance on maternally derived antioxidants [8].

Obesity in pregnancy is characterized by chronic low-grade inflammation, altered mitochondrial function, and increased systemic oxidative stress [9]. Although oxidative stress has been implicated in later pregnancy complications such as preeclampsia and fetal growth restriction [2], its role in early gestation, particularly in the context of maternal obesity and metabolic stress, remains underexplored. Emerging data suggest that disruption of redox homeostasis during the FT may impair placentation and contribute to adverse pregnancy outcomes and long-term developmental programming of the offspring [10,11].

Whether maternal obesity perturbs this balance at the transcriptional, translational or post-translational level in the FT remains unclear [12]. Evidence from human FT placental studies is limited and contradictory, with some investigations reporting increased oxidative stress or antioxidant gene activation in maternal obesity in FT placenta [13], while others detect no significant oxidative damage [14,15]. Such discrepancies may reflect differences in gestational age, maternal perfusion dynamics or compensatory responses. They may also arise because activation of stress pathways is not captured by conventional oxidative damage markers [16].

Beyond changes in oxidative balance, maternal metabolism is altered in obesity already during the FT [17,18], and these changes are accompanied by alterations in the placental transcriptome and proteome in FT placentas [17,19]. Functionally, an imbalance in cytotrophoblast turnover has been reported, characterized by increased apoptosis and reduced proliferation [15]. This may result from elevated DNA damage [15] and impaired DNA damage repair [19,20]. Oxidative stress is a well-established inducer of DNA damage, affecting both DNA bases and the backbone [20]; however, evidence of oxidative DNA damage has not been detected in FT placentas from obese women [15]. Consistent with this, no changes were observed in the oxidative stress-sensitive markers HO-1 and Hsp70 [15]. These observations raise the possibility that early placental stress in maternal obesity may involve mechanisms and targets distinct from classical oxidative injury.

Here, we tested the hypothesis that maternal overweight and obesity are associated with oxidative damage in the FT placenta and sought to determine whether early placental stress responses are accompanied by transcriptional remodeling of antioxidant defense and stress signaling pathways. We therefore aimed to quantify oxidative lipid and protein modifications and analyze the expression of key antioxidant enzymes and stress-responsive genes, focusing on the glutathione- and thioredoxin-dependent systems that constitute the principal redox buffering mechanisms in early placental development [8,21]. Improved understanding of these early placental responses may provide mechanistic insight into placental adaptation to maternal obesity and inform future strategies to improve pregnancy and offspring health.

## 2. Materials and Methods

### 2.1. Ethical Approval

This study was approved by the institutional review board and ethical committee of the Medical University of Graz (#29-095 ex16/17; Date: 23 December 2016) and was performed in accordance with the latest revision of the Declaration of Helsinki. Women with a singleton pregnancy scheduled for legal elective pregnancy termination were recruited upon signing written informed consent. Patient details were collected at admission and were limited to those listed in Table 1 and below (c.f. Tissue collection) to protect patient privacy as a requirement of ethical approval.

Following the Shapiro–Wilk test for normality, data were compared using Student’s *t*-test. Data are given as mean ± sd. BMI, body mass index; 4-HNE, 4-Hydroxynonenal. Lean refers to samples with BMI < 25 kg/m^2^ and overweight and obese with BMI ≥ 25 kg/m^2^. The gestational ages of the samples included in this study ranged from 4 + 0 to 12 + 0 weeks of gestation. Samples used for nitrotyrosine immunoblot are the same as for 4-HNE. One sample from a lean woman used for 4-HNE was exhausted and could not be included in the nitrotyrosine analysis.

### 2.2. Tissue Collection

Human FT placental tissue (gestational weeks 5–12 post menstruation, p.m.) was obtained after voluntary termination. In total, *n* = 101 samples were collected, with the specific numbers used for each experiment depicted in Table 1. Exclusion criteria were smoking, assessed by questionnaire and verified by serum cotinine concentrations using a cut-off >4.47 ng/mL [22], other co-morbidities and current medication. Body mass index (BMI), calculated from body weight (kg) measured at time of pregnancy termination and height (m), ranged from 19.2 to 39.5 kg/m^2^. Gestational age was calculated based on women’s last menstrual period and verified by measurement of fetal crown–rump length at the time of pregnancy termination. Cases with gestational age difference between last menstrual period and fetal crown–rump length > 6 days were excluded. Tissue was washed with phosphate buffered saline (PBS, Sigma Aldrich, St. Louis, MO, USA) and cryopreserved at −80 °C after snap-freezing until further use. The time between tissue collection and cryopreservation was recorded (“preparation time”) and used as a covariate [23].

### 2.3. First-Trimester Chorionic Villus Explant Culture

Human FT chorionic villi from lean women (*n* = 8) were micro-dissected (15–20 mg wet weight), rinsed with PBS and cultured in Dulbecco’s Modified Eagle Medium (DMEM; Gibco, Invitrogen, Carlsbad, CA, USA) and Ham’s F-12 medium 1:1 (*v*/*v*; Gibco) supplemented with 10% fetal calf serum (FCS, Thermo Scientific, Rockford, IL, USA) and 1% penicillin–streptomycin (Gibco). Explants were pre-conditioned at 2.5% O_2_ on a hypoxic workstation (BioSpherix; Redfield, NY, USA) for 24 h and treated with TNF-α (50 ng/mL, Sigma Aldrich) at 2.5% O_2_ for 48 h to mimic long-term exposure to the inflammatory environment. The TNF-α concentration and 48 h exposure were selected based on previously established protocols using FT human placental villous explants, where this regimen reproducibly induces sustained inflammatory responses while maintaining tissue viability [24,25,26]. The effect of oxygen tension was additionally assessed at 6.5% O_2_ for 48 h. Explants were snap-frozen for subsequent RNA extraction or protein isolation. Culture supernatants were frozen and used for human ß-chorion gonadotropin quantification (IMMULITE 1000 Systems Immunoassay, Siemens, Munich, Germany).

### 2.4. RNA Isolation and Reverse Transcription

FT placental tissue was homogenized in RLT Plus Buffer (Qiagen, Hilden, Germany) with 1% (*v*/*v*) β-mercaptoethanol (Merck, Darmstadt, Germany) using a tissue lyzer (MagNa Lyser, Roche, Basel, Switzerland). Total RNA was isolated with the AllPrep DNA/RNA/miRNA Universal Kit (Qiagen) according to the manufacturer’s guidelines. mRNA quality was controlled using Bioanalyzer (Agilent, Santa Clara, CA, USA) with an inclusion cutoff RNA integrity number (RIN) ≥ 3. Three samples with RIN < 3 were not included in further analyses. RIN values (mean ± SD) of all samples for Nanostring analyses were 7.1 ± 1.0. After quality control, mRNA was reversely transcribed using a SuperScript II Reverse Transcriptase kit (Life Technologies, Carlsbad, CA, USA) as per the manufacturer’s protocol.

### 2.5. Nanostring

Expression of genes involved in antioxidant defense and placental stress signaling was quantified using a NanoString nCounter system (Nanostring Technologies, Seattle, WA, USA), which is based on the digital detection of mRNA molecules using a color-coded probe pair that specifically hybridize to target molecules. Gene expression was measured by counting the barcode for each specific molecule, which is detected by a digital analyzer. Probe pairs were synthesized at Integrated DNA Technologies (Leuven, Belgium). Positive normalization to the geo-mean of the top three positive controls and codeset normalization [27] to reference genes WD Repeat Domain 45B (*WDR45L*) and TATA-binding protein (TBP) were performed by nSolver 4.0 software (Nanostring Technologies). Unlike traditional reference genes such as *GAPDH* and *ACTB*, which we and others have found to be inferior to *TBP* for normalization in human placenta, *WDR45L* was additionally selected based on our own validation tests across various placental tissues and cell lines [28]. Results are expressed as gene counts of mRNA molecules in 100 ng/µL RNA.

### 2.6. Protein Isolation, Quantification and Immunoblotting

FT placental tissue was homogenized in RIPA buffer (Sigma Aldrich) with protease inhibitors (Roche, Basel, Switzerland). The protein amount was quantified using a BCA assay (Thermo Scientific). Protein lysates were mixed with Laemmli buffer 2x (Sigma Aldrich) and denatured for 5 min at 96 °C. Total protein (10 µg) was loaded onto 4–20% SDS-PAGE gels (BioRad Laboratories, Hercules, CA, USA), separated at 120 V for 1 h, and transferred onto nitrocellulose membranes using a TurboBlot system (BioRad). Blocking was performed for 1 h with 5% non-fat dry milk (BioRad) in tris-borate-EDTA (TBE) + 0.1% Tween 20 (Sigma Aldrich). Membranes were incubated with primary antibodies against 4-Hydroxynonenal (Abcam, Cambridge, UK, ab48506) at dilution 1:100 or 3-nitrotyrosine (Cell Signaling, Danvers, MA, USA, #9691) at dilution 1:1000 overnight at 4 °C. Thereafter, membranes were incubated with HRP-conjugated secondary antibody (1:2000, BioRad) for 1 h at room temperature. For normalization and loading control, the same membranes were incubated with antibody against β-actin (Abcam, ab8227) at dilution 1:1000. Inter-gel variability was controlled by running one identical sample, which was present on all membranes testing the same target. Immunoreactivity was visualized using a SuperSignal-Pico Chemiluminescent Substrate (Thermo Scientific) and with a Fusion FX system (Vilber Lourmat, Eberhardzell, Germany). Band intensities were quantified using EvolutionCapt software v18.12 (Vilber Lourmat, Eberhardzell, Germany). To quantify the overall level of oxidative modification, integrated densitometry was performed across the entire immunoreactive region between approximately 25 and 100 kDa for 4-HNE and nitrotyrosine immunoblots, respectively [29,30]. Results are presented as the ratio of band densities of target protein and reference protein. Samples used for nitrotyrosine immunoblotting are the same as for 4-HNE. One sample from a lean woman used for 4-HNE was exhausted and could not be included in the nitrotyrosine analysis.

### 2.7. Statistical Analysis

For statistical analysis, IBM SPSS Statistics 25 (Armonk, NY, USA) and GraphPad Prism 10 (Boston, MA, USA) were used. The normal distribution of data was tested using the Shapiro–Wilk test. Associations between maternal BMI and experimental outcomes were assessed using multivariate linear regression models with adjustments for gestational age and preparation time. Gestational age and preparation time were considered continuous variables. Maternal BMI was used as a continuous variable for the statistical analyses but was categorized into lean (BMI < 25 kg/m^2^) vs. overweight (BMI 25–30 kg/m^2^) and obese (BMI ≥ 30 kg/m^2^) for graphical representation. As determined by multivariate analysis, maternal age (*p* = 0.323) and fetal sex (*p* = 0.439) had no effect on the results and were not included in the final model as confounders. *p* <  0.05 was considered statistically significant.

## 3. Results

### 3.1. Lipid Peroxidation and Protein Nitration

We have previously established that obesity in pregnancy is characterized by the absence of oxygen-induced DNA damage in FT placenta [15]. This raised the hypothesis that other established oxidative stress targets, such as lipids and proteins, would be modified by oxidative stress in the FT of women with obesity. To further characterize the redox status of the FT placenta in maternal overweight and obesity, we quantified markers of lipid peroxidation and protein nitration in placental tissue from lean vs. overweight and obese women [29].

**Figure 1:**
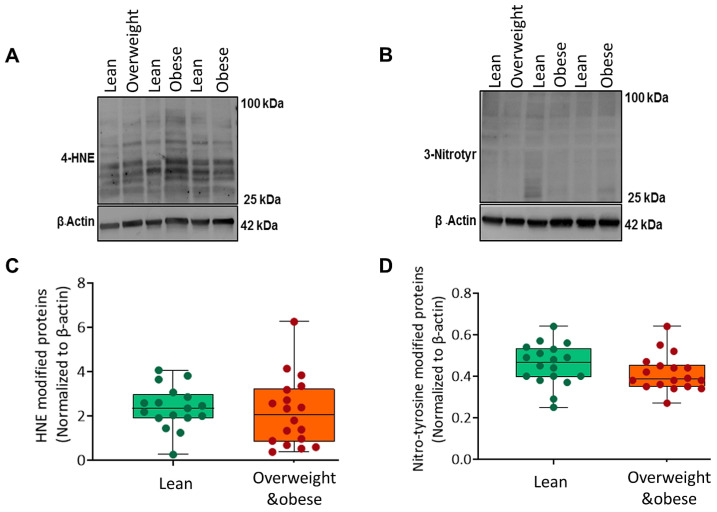
Lipid peroxidation adducts (4-HNE) and protein tyrosine nitration (3-Nitrotyr) in FT placental tissue. 4-HNE and 3-Nitrotyr protein levels were determined in FT placental tissue from lean (4-HNE: *n* = 21, 3-Nitrotyr: *n* = 19) and overweight and obese (4-HNE: *n* = 18, 3-nitrotyrosine: *n* = 17) women by Western Blotting (**A**,**B**). For global assessment of protein lipid peroxidation and nitration, immunoblots for 4-HNE (**C**) and 3-Nitrotyr (**D**) were quantified by integrated densitometry across the entire immunoreactive region between approximately 25 and 100 kDa. β-actin was used for normalization as a loading control. The band range from 25 to 100 kDa is indicated where the strongest signal is expected. Data show the mean ± SD. Statistical analysis used a multivariate linear model with BMI as the continuous dependent variable and adjustment for gestational age and tissue processing time. The figures show the results for both ends of the BMI continuum. Gestational weeks 5–12. Samples used for nitrotyrosine immunoblot are the same as for 4-HNE. One sample from a lean woman used for 4-HNE was exhausted and could not be included in the nitrotyrosine analysis.

To this end, we quantified 4-hydroxynonenal (4-HNE) and 3-nitrotyrosine (3-Nitrotyr)-modified proteins as markers of redox imbalance and oxidative stress in the FT placenta by Western blotting. No difference in 4-HNE (Figure 1A,C) and 3-Nitrotyr adduct levels (Figure 1B,D) was observed in protein lysates of FT placental tissue from lean vs. overweight and obese women.

### 3.2. Antioxidant Defense in FT Placenta of Women with Obesity

Our previous findings of absent oxidative DNA damage and unaltered classical stress markers (HSP70, HO-1) [15], as well as findings here of unaltered oxidative damage of lipids and proteins (4-HNE and 3-Nitrotyr), may be underpinned by increased placental antioxidant defense in maternal overweight and obesity. We tested this by analyzing gene expression on an extensive set of enzymes involved in the maintenance of redox homeostasis known to be functionally relevant in early first-trimester placental tissue [8,21]. Nanostring analysis of 17 antioxidant defense enzymes showed that expression of 5 FT placental genes was decreased in obesity cases: Microsomal glutathione S-transferase 3 (*MGST3*, −11.2%, *p* = 0.01), Superoxide dismutase 3 (*SOD3*, −30.2%, *p* = 0.02), Glutathione S-transferase P (*GSTP1*, −9.7%, *p* = 0.02), Glutaredoxin (*GLRX*, −17.9%, *p* = 0.04) and Thioredoxin reductase 1 (*TXNRD1*, −11.0%, *p* = 0.04). All other antioxidant defense genes were unaltered (Table 2).

**Figure 2:**
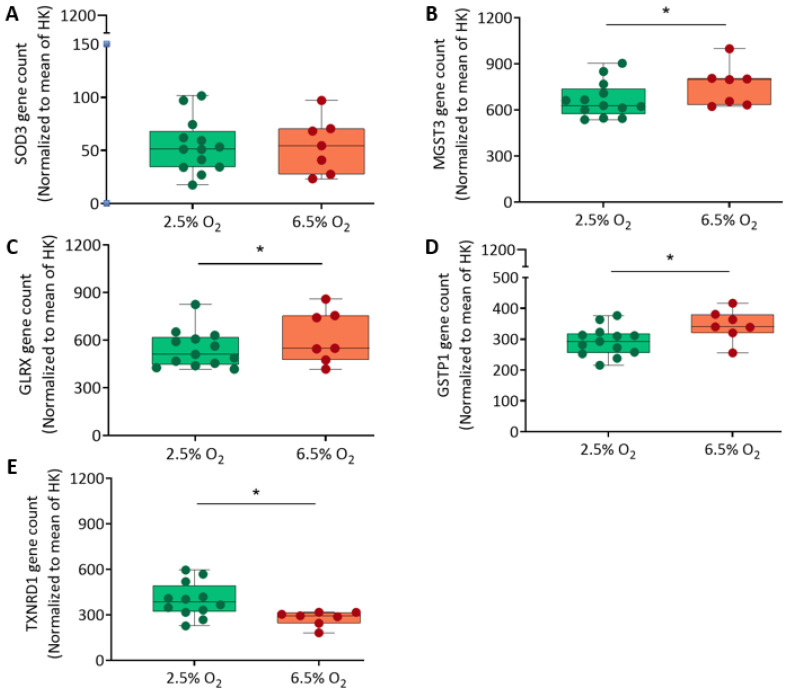
Effect of oxygen tension on antioxidants altered by maternal overweight and obesity after exposure of first-trimester (FT) placental chorionic villous explants to physiologically lower (2.5% O_2_) and higher (6.5% O_2_) oxygen levels. FT chorionic villous explants from placental tissues of lean women (*n* = 8, gestational week 5–10) were cultured for 48 h in triplicates at 2.5% O_2_ or 6.5% O_2_, respectively. Gene expression of *SOD3* (**A**), *MGST3* (**B**), *GLRX* (**C**), *GSTP1* (**D**) and *TXNRD1* (**E**) was analyzed by Nanostring gene expression analysis. Data was normalized to the mean of two different housekeeping (HK) genes: WD repeat domain 45B (*WDR45L*) and TATA box binding protein (*TBP*). Statistical analysis included paired *t*-test or Wilcoxon matched-pairs signed rank test as appropriate. * *p* < 0.05.

One reason for lower expression of antioxidative defense genes could be the lack of a physiological stimulus, i.e., absence of increased oxygen tension in the intervillous space in obesity. We tested this hypothesis in vitro by examining whether the antioxidant defense enzymes altered in maternal overweight and obesity respond to changes in oxygen tension. To this end, we cultured FT chorionic villous explants from lean women under lower (2.5% O_2_) and higher (6.5% O_2_) oxygen tension, both physiological in the FT intervillous space [31]. Exposure of FT chorionic villous explants did not affect the expression of *SOD3* (Figure 2A). However, *MGST3* (14.1%, *p* = 0.02), *GLRX* (14.1%, *p* = 0.02), and *GSTP1* (17.8%, *p* = 0.02) gene expression was induced by raising oxygen tension to 6.5% O_2_ (Figure 2B–D). Hence, the placental antioxidative defense systems measured here respond to rising oxygen tension by increasing their expression level. These results support the notion that the lower antioxidative defense systems associated with maternal overweight and obesity in FT placentas are the result of a lack of rising oxygen tension in the intervillous space. Among antioxidant defense enzymes altered in obesity, only *TXNRD1* (30.1%, *p* = 0.05) was downregulated by 48 h exposure to high oxygen levels in vitro (Figure 2E).

### 3.3. FT Placental Stress Signaling in Obesity

A further reason for absence of oxidative damage in FT placental tissues might be altered—potentially defective—stress signaling. To test this hypothesis, we selected a set of prominent and well-established stress signaling molecules and compared their gene expression levels between lean and overweight and obese FT placentas by Nanostring analysis.

Among nine targets that we surveyed, we found higher levels of Mitogen-Activated Protein Kinase Kinase Kinase 5 (*MAP3K5*, alias: *ASK1*) and Mitogen-Activated Protein Kinase 11 (*MAPK11*) and lower levels of Mitogen-Activated Protein Kinase 13 (*MAPK13*), Mitogen-Activated Protein Kinase 12 (*MAPK12*), and Mitogen-Activated Protein Kinase 8 (*MAPK8*, alias: *JNK1*) in the FT placental samples from overweight and obese as compared to lean women. Two transcriptional master regulators, i.e., Nuclear factor erythroid 2-related factor 2 (*NFE2L2*, alias: Nrf2) and Hypoxia-inducible factor 1-alpha (*HIF1A*), were not altered in maternal overweight and obesity (Table 3).

These data indicate that maternal overweight and obesity are associated with a transcriptional shift in FT placenta stress signaling without influencing the canonical effectors *Nrf2* and *HIF1A*.

Since genes with significantly altered expression levels in maternal overweight and obesity, i.e., *ASK1*, *MAPK11*, *MAPK12*, *MAPK13* and *JNK 1*, belong to the TNF-α signaling pathway [32], we further investigated the potential of long-term inflammatory stress to induce changes in cellular stress markers. With long-term exposure of lean FT placental explants, we aimed to mimic the chronic low-grade inflammation present already in early pregnancy of women with obesity [33]. To this end, we cultured FT chorionic villous explants at 2.5% oxygen with TNF-α (50 ng/mL) for 48 h and measured those classical stress response markers that had been significantly changed in overweight and obesity (Table 3).

**Figure 3:**
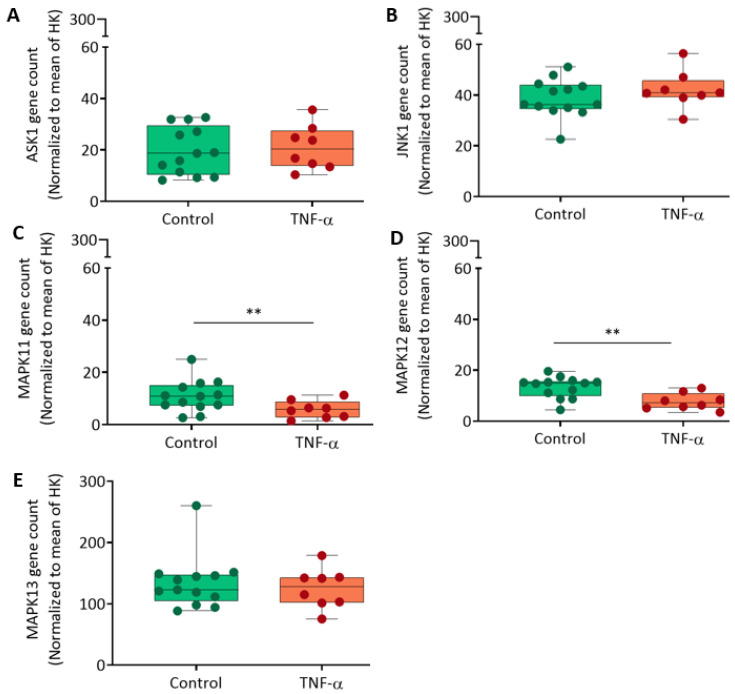
Assessment of overweight and obesity-affected stress signaling genes after treatment with tumor necrosis factor α (TNF-α) in FT placental explants of lean women. FT chorionic villous explants from different placental tissues (*n* = 8, gestational weeks 5–10) were cultured at 2.5% O_2_ with TNF-α (50 ng/mL) for 48 h in triplicate. Gene expression of *ASK1* (**A**), *JNK1* (**B**), *MAPK11* (**C**), *MAPK12* (**D**) and *MAPK13* (**E**) was analyzed by Nanostring gene expression analysis. Data was normalized to the mean of two different housekeeping genes, namely WD repeat domain 45B (*WDR45L*) and TATA box binding protein (*TBP*). Statistical analysis included Paired *t*-test or Wilcoxon matched-pairs signed rank test as appropriate. ** *p* < 0.01.

Only *MAPK11* and *MAPK12* were downregulated by TNF-α in FT chorionic villous explants by 46.7% and 42.2% (*p* < 0.01), respectively. All other genes altered in FT total tissue with obesity, i.e., *ASK1*, *JNK1* and *MAPK13*, were not affected by long-term (48 h) treatment with TNF-α in vitro (Figure 3A–D).

**Figure 4:**
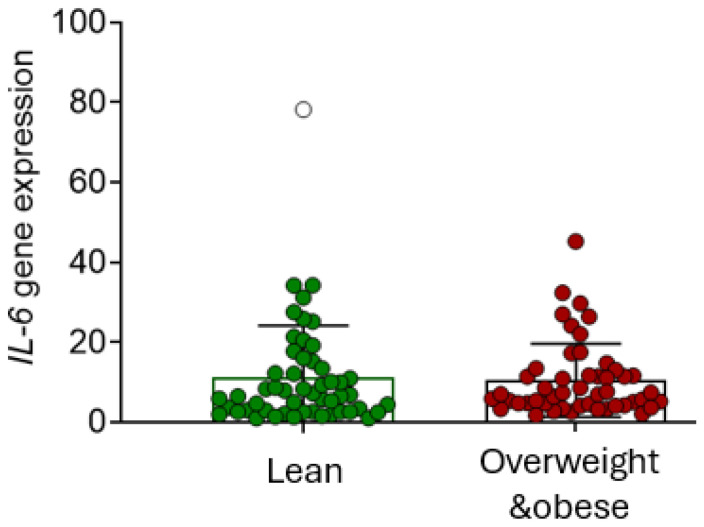
Gene expression of *IL6* was analyzed by Nanostring gene expression analysis. Data was normalized to the mean of two different housekeeping (HK) genes: WD repeat domain 45B (*WDR45L*) and TATA box binding protein (*TBP*). Statistical analysis used a multivariate linear model with BMI as continuous dependent variable and adjustment for gestational age and tissue processing time. Gestational weeks 5–12.

The FT of pregnancies with obesity is characterized by low-grade inflammation, as demonstrated by increased circulating TNF-α levels [34]. This led us to speculate that consequential inflammatory stress in placental tissue may account for the low *MAPK11* and *MAPK12* levels in FT placentas of women with overweight and obesity. Therefore, we determined the expression of *IL6* gene, a pro-inflammatory marker whose expression is associated with metabolic inflammation in obesity [35]. There was no difference between FT placental tissue of lean vs. overweight and obese women (Figure 4).

### 3.4. Oxidative Damage Is Not Associated with Expression of Antioxidant Enzymes and Stress Signaling-Related Genes

**Figure 5:**
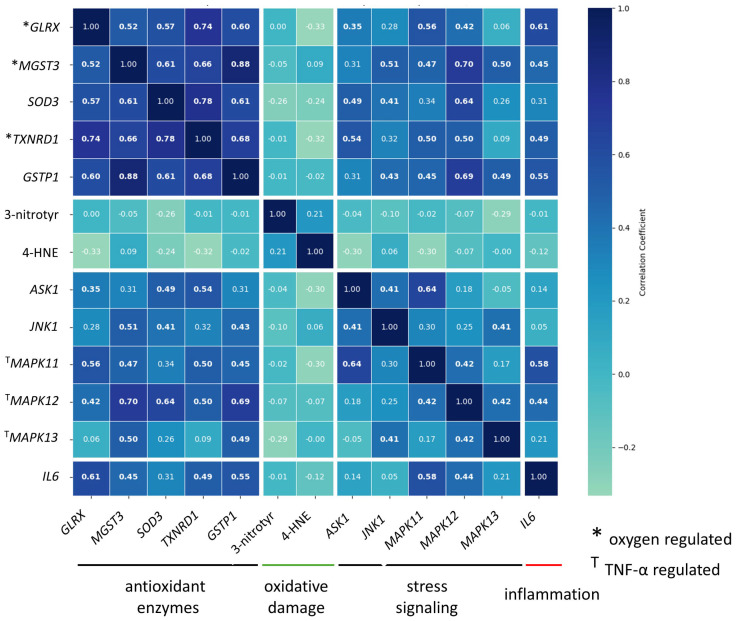
Correlation heatmap of inflammation and oxidative damage markers with the expression of antioxidant and stress-related genes in first-trimester (FT) placenta. Oxidative damage markers of lipid peroxidation and protein nitration were measured by Western blotting, and *IL6* expression by Nanostring. Heatmap was constructed using Spearman correlation coefficients, with blue indicating stronger positive correlations and yellow weaker negative correlations. * depicts oxygen- and ^T^ denotes TNF-α-regulated genes, as demonstrated in the explant experiments. Numbers in bold indicate significant correlation with *p* < 0.05.

To explore whether specific biological stressors may be driving the selective transcriptional responses observed in the FT placenta of overweight and obese women, we performed correlation analysis among antioxidant enzymes, stress signaling genes, and markers of oxidative damage and inflammation. This approach aimed to assess whether expression of key stress defense enzymes and stress pathway components aligned with oxidative injury or inflammatory activation across samples. We found strong positive correlations among several antioxidant genes (e.g., *GLRX*, *TXNRD1*, *SOD3*) and between these genes and stress-responsive MAPK family members (*MAPK11*, *MAPK12*), suggesting a coordinated redox-stress transcriptional response. Notably, *IL6* expression, a marker of inflammation, positively correlated with multiple antioxidant enzymes and MAPK signaling genes, including *MAPK11* and *MAPK12*. In contrast, markers of oxidative damage, 4-HNE and 3-nitrotyrosine, showed no significant correlations with antioxidant or stress-related gene expression (Figure 5).

Overall, our findings indicate that transcriptional regulation of antioxidant defense enzymes and cellular stress markers in the FT placenta of overweight and obese women is linked more strongly to inflammatory signaling than to oxidative damage.

## 4. Discussion

In the first trimester of human pregnancy, placental antioxidant defenses are low and increase mainly toward the end of this period, potentially heightening susceptibility to oxidative stress [6]. However, our previous work found no evidence of obesity-associated oxidative DNA damage (8-OHdG) or of induction of classical stress markers (HSP70, HO-1) in FT tissue [15]. Building on these findings, we asked whether maternal overweight and obesity are associated with oxidative stress and altered transcription of antioxidant enzymes and stress-responsive signaling genes in the FT placenta.

The key findings are (i) there is no evidence for overweight and obesity-associated oxidative stress-induced lipid and protein damage in bulk analysis of whole FT placental tissue, (ii) maternal overweight and obesity are associated with transcriptional downregulation of specific antioxidant defense enzymes, (iii) a shift in expression of cellular stress markers indicates transcriptional remodeling of MAPK-related genes, and (iv) inflammatory cues reflected by *IL6* gene expression rather than oxidative protein damage (4-HNE and nitrotyrosine modifications) associate with transcriptional activity of antioxidant defense enzymes and cellular stress markers. Together, these data indicate that maternal overweight and obesity are linked to an altered transcriptional landscape of antioxidants and stress response without overt oxidative injury in FT placenta.

Obesity *per se* is linked to hyperglycemia-induced systemic oxidative stress [36]. In such an environment, highly reactive ROS can further react with biological molecules, such as DNA, lipids and proteins, leading to the formation of 8-oxo-7,8-dihydroguanine (8-OHdG), lipid peroxidation and protein nitration [37,38]. In line with our previous study, where we established the absence of oxidative DNA damage in early placenta complicated by maternal obesity, we now report the absence of lipid and protein modifications, as there was no difference in 4-HNE and nitrotyrosine levels in FT placenta between lean vs. overweight and obese women. Specifically, 29% of antioxidant genes analyzed i.e., *MGST3*, *SOD3*, *GSTP1*, *GLRX* and *TXNRD1*, were downregulated in maternal overweight and obesity in early placenta, whereas others were unaltered. Notably, differently expressed genes are mostly components of glutathione- and thioredoxin-dependent detoxification systems and extracellular redox regulation [39]. *MGST3* and *GSTP1* participate in glutathione conjugation of electrophilic lipid peroxidation products [40], *GLRX* mediates protein deglutathionylation [41], and *TXNRD1* sustains cytosolic thioredoxin recycling [39]. *SOD3* regulates extracellular superoxide detoxification and nitric oxide bioavailability [42]. In contrast, genes involved in mitochondrial ROS detoxification (*SOD2*, *PRDX3*, *TXNRD2*) [43], core glutathione synthesis and recycling (*GSS*, *GSR*), and peroxide reduction (*GPX1–3*) [44] were unchanged, as was the ROS-generating enzyme *NOX1* [45]. This pattern suggests selective modulation of downstream redox buffering and signaling pathways rather than global activation of ROS production or mitochondrial oxidative stress.

Under physiological conditions, FT placentas from lean women can upregulate expression of antioxidant enzymes such as *MGST3*, *GSTP1*, and *GLRX* in response to the physiological rise in oxygen tension, as demonstrated in placental explants exposed to higher, yet physiological, oxygen concentrations. The oxygen exposure experiments were designed to determine whether the genes found to be differentially expressed in vivo are intrinsically responsive to physiologically relevant changes in oxygen levels characteristic of early placental development. They demonstrate that modulation of oxygen tension *per se* can regulate selected antioxidant transcripts (e.g., *MGST3*, *GSTP1*, and *GLRX*), while *SOD3* and *TXNRD1* did not show altered expression and seem to be regulated by other factors, e.g., cytokines, growth factors and Nrf2-dependent signaling. Indeed, also in vivo, in normal pregnancy, placental SOD1 and SOD2, catalase, and glutathione peroxidase expression and activity steeply rise between 8 and 12 weeks, coinciding with early oxygen tension changes [6]. Therefore, the absence of overt oxidative damage despite reduced expression of antioxidant defense genes is consistent with the possibility that oxygen tension rises less steeply in the intervillous space, potentially owing to delayed spiral artery opening in maternal obesity [46]. Notably, the negative correlation between oxidative stress and gestational age observed in normal FT placenta is absent in corresponding obese FT tissue [13].

In a well-oxygenated environment, hyperglycemia is a key driver of oxidative stress through stimulating oxidative metabolism. However, obesity is not associated with maternal hyperglycemia in the FT [47]. Alternatively, a lipotoxicity-induced, pro-inflammatory environment may underpin changes observed in pregnant women with obesity [42,48,49]. Indeed, reduced expression of fatty acid oxidation and esterification genes [50], accompanied by elevated triglycerides [51], was reported in FT placentas in obesity—changes which persist to term [49,52]. Recently, we have demonstrated that inflammatory, not oxidative, stress is associated with increased expression of DNA damage and repair genes in FT placenta [15]. Here, we measured the expression of classical stress-responsive genes and found obesity-associated downregulation of *MAPK12*, *MAPK13* and *JNK1* in FT placenta and upregulation of *ASK1* and *MAPK11*. To further explore whether inflammatory signaling could contribute to this transcriptional pattern, we exposed FT placental explants from lean women to the pro-inflammatory cytokine TNF-α under physiological oxygen tension. The 48 h exposure period was chosen to model the sustained low-grade inflammatory environment characteristic of maternal obesity rather than to capture acute kinase activation events [24,25,26]. Under these conditions, *MAPK11* and *MAPK12* were responsive to TNF-α stimulation, indicating that prolonged cytokine exposure can modulate stress-related transcription in placental tissue. The absence of a significant difference in mean *IL6* expression between BMI groups does not preclude biologically meaningful associations. Correlation analyses capture continuous relationships independent of group comparisons. Notably, *IL6* expression, an established marker of inflammation [53], positively correlated with multiple antioxidant enzymes and MAPK signaling genes, including *MAPK11* and *MAPK12*. In contrast, the markers of oxidative damage 4-HNE and 3-nitrotyrosine showed no correlations with antioxidant or stress-related gene expression. These associations do not prove causality but suggest that inflammatory signaling is more closely linked to transcriptional remodeling than measurable oxidative damage within the parameters assessed in this study. Importantly, inflammatory and oxidative stress cascades are mechanistically interconnected through redox-sensitive transcription factors and kinase networks and cannot be considered fully independent. Thus, rather than indicating dissociation of inflammatory and antioxidant pathways, our findings support a model in which sustained low-grade inflammation in maternal overweight and obesity contributes to modulation of stress and antioxidant gene expression in early placental tissue, without overt oxidative damage. Consistent with this concept, MAPK-signaling pathways have also been implicated in lipotoxicity-related stress responses in obesity in third-trimester placenta mediated by early growth response protein-1 [54].

These findings further strengthen the hypothesis that in the intrauterine environment associated with overweight and obesity, low-grade inflammation rather than oxidative stress act as the primary stimulus for transcriptional activation of antioxidant and stress signaling programs already in the early placenta.

### Strengths and Limitations

This study was made possible by our unique FT cohort, which provides placental tissue spanning a broad range of maternal BMI and gestational ages (weeks 5–12). To minimize confounding from tobacco exposure, we rigorously excluded smokers using cotinine measurements in combination with self-reported information, as smoking is known to induce oxidative stress even in FT placenta [55].

A major consideration in FT placental research is the physiological rise in oxygen tension within the intervillous space and placental tissue as pregnancy progresses, a process that is essential for normal development and trophoblast function [6,21]. Accordingly, gestational age was defined *a priori* as a key confounder and included in all statistical analyses. In addition, all tissue culture experiments were performed under physiologically low oxygen tension (2.5% O_2_) to avoid potential hyperoxic effects associated with ambient oxygen.

Although the cohort covered a broad gestational age window, the sample size within individual weeks was not sufficient to robustly stratify the dataset and analyze discrete FT periods separately. Therefore, adjusting for gestational age was the most appropriate approach to account for developmental changes across weeks 5–12 while retaining statistical power. For the same reason, we were unable to stratify the dataset according to fetal sex.

It is important to note that transcriptional changes do not necessarily reflect functional pathway activation. Several stress regulators, including HIF1α and MAP kinases, are predominantly controlled at the post-translational level through protein stabilization or phosphorylation [56,57]. Therefore, while our data demonstrate transcriptional remodeling, they do not directly assess kinase activation or protein-level dynamics. Future studies incorporating proteomic and phospho-protein analyses will be necessary to further elucidate functional pathway regulation in early placental tissue. Furthermore, we performed our analyses in total placental tissue and, thus, cannot exclude cell-specific oxidative changes. Finally, placental tissue was obtained from pregnancy terminations for psychosocial (i.e., non-medical) reasons; thus, pregnancy outcome data is unavailable, and the presence of unrecognized genetic abnormalities in the tissue cannot be excluded.

Although our findings provide mechanistic insight into early placental adaptation to maternal overweight and obesity, whether these transcriptional changes influence placental function or subsequent pregnancy and offspring outcomes remains to be determined. Future studies integrating molecular, functional, and longitudinal clinical data are needed to establish their clinical significance.

## 5. Conclusions

Our findings support the presence of adaptive responses in the FT placenta in pregnancies complicated by maternal overweight and obesity. These adaptations are characterized by altered expression of antioxidant and stress response genes and by changes in stress signaling pathways that were more closely associated with *IL6* expression than with markers of lipid or protein oxidation. Together with the previously reported absence of detectable oxidative DNA damage, these findings suggest that the FT placenta adapts to a mildly pro-inflammatory and pro-oxidant environment before overt oxidative injury develops. Such adaptations may reflect exposure to moderately elevated ROS levels already present before conception in women with overweight or obesity. Alternatively, they may result from delayed spiral artery remodeling, leading to a postponed rise in intervillous oxygen tension during early placental development. Future studies integrating transcriptomic, proteomic, and enzymatic activity analyses of ROS-generating and antioxidant defense pathways will be important to determine whether these transcriptional changes translate into functional alterations in placental redox homeostasis.

## Figures and Tables

**Table 1:** Description of the study cohorts by experiment for the respective groups, lean vs. overweight and obese.

Experiment	Characteristics	Maternal BMI (kg/m^2^)	Gestational Age (Days)	Maternal Age (Years)
Nanostring	Lean (*n* = 53)	22.1 ± 1.5	55.7 ± 14.3	30.1 ± 8.7
	Overweight and obese (*n* = 48)	29.8 ± 4.3	51.6 ± 15.0	31.3 ± 6.5
	*p*-value	<0.0001	0.170	0.438
4-HNE immunoblot	Lean (*n* = 18)	21.8 ± 1.5	53.0 ± 14.5	30.7 ± 5.9
	Overweight and obese (*n* = 34)	30.9 ± 4.3	52.3 ± 14.7	30.6 ± 6.3
	*p*-value	<0.0001	0.930	0.418
Nitrotyrosine immunoblot	Lean (*n* = 17)	21.6 ± 1.1	51.9 ± 14.3	31.2 ± 6.0
	Overweight and obese (*n* = 34)	31.0 ± 4.5	52.3 ± 14.7	30.6 ± 7.3
	*p*-value	<0.0001	0.921	0.349

**Table 2:** Gene expression of antioxidant defense enzymes in FT tissue from lean vs. overweight and obese women.

	Unstandardized Coefficients		Standardized Coefficients	t	Sig.
Gene	B	Std. Error	Beta		
***MGST3***	−172.571	69.183	−0.241	−2.494	**0.014**
***SOD3***	−25.233	10.415	−0.199	−2.423	**0.017**
***GSTP1***	−48.850	20.811	−0.223	−2.347	**0.021**
***GLRX***	−334.134	156.636	−0.215	−2.133	**0.035**
***TXNRD1***	−37.285	18.253	−0.203	−2.043	**0.044**
*PRDX3*	−104.245	61.033	−0.173	−1.708	0.091
*SOD1*	−5.765	3.500	−0.157	−1.647	0.103
*SOD2*	131.642	83.597	0.153	1.575	0.119
*SRXN1*	−9.674	6.594	−0.145	−1.467	0.146
*GPX3*	309.538	217.737	0.145	1.422	0.158
*TXNRD2*	−6.852	5.151	−0.128	−1.330	0.187
*GPX1*	−18.232	15.332	−0.113	−1.189	0.237
*GPX2*	−4.367	3.687	−0.121	−1.185	0.239
*GSS*	−4.992	5.302	−0.092	−0.942	0.349
*GSR*	13.764	21.807	0.056	0.631	0.529
*TXN*	−16.333	33.926	−0.048	−0.481	0.631
*NOX1*	−0.230	1.856	−0.013	−0.124	0.902

Dependent variable: gene; multivariate linear regression model with BMI as continuous variable, with lean as reference, and with adjustment for gestational age and tissue processing time. Significant genes are shown in bold. *n* = 101. Gestational weeks 5–12.

**Table 3:** Gene expression of selected cellular stress signaling genes.

	Unstandardized Coefficients		Standardized Coefficients	t	Sig.
Gene	B	Std. Error	Beta		
***ASK1***	24.924	12.105	0.192	2.059	**0.042**
***MAPK11***	2.877	1.437	0.195	2.003	**0.048**
***MAPK13***	−20.798	8.486	−0.242	−2.451	**0.016**
***MAPK12***	−5.623	2.299	−0.236	−2.445	**0.016**
***JNK1***	−6.176	2.774	−0.203	−2.226	**0.028**
*JNK2*	−8.604	8.264	−0.106	−1.041	0.300
*Nrf2*	11.741	12.818	0.084	0.916	0.362
*HIF1A*	17.727	24.575	0.071	0.721	0.472
*MAPK14*	−1.640	21.696	−0.007	−0.076	0.940

Dependent variable: gene; multivariate linear regression model with BMI as continuous variable and adjustment for gestational age and tissue processing time. Significant genes are shown in bold. *n* = 101. Gestational weeks 5–12.

## Data Availability

The original contributions presented in this study are included in the article. Further inquiries can be directed to the corresponding authors.
